# The Newton-X platform for mixed quantum–classical dynamics

**DOI:** 10.1039/d6cp01391k

**Published:** 2026-06-04

**Authors:** Mario Barbatti, Rafael S. Mattos, Baptiste Demoulin, Matheus de O. Bispo, Mattia Bondanza, Marcus Brady, Rachel Crespo-Otero, Ely G. F. de Miranda, Pavlo O. Dral, Giovanni Granucci, Anna Hehn, Federico J. Hernández, Gabriele Iuzzolino, Ritama Kar, Fábris Kossoski, Hans Lischka, Benedetta Mennucci, Saikat Mukherjee, Anik Mukhopadhyay, Fulvio Perrella, Maurizio Persico, Max Pinheiro, Jiri Pittner, Felix Plasser, Nadia Rega, Eduarda Sangiogo-Gil, Tejas Thorat, Josene M. Toldo, Anderson A. Tomaz, Márcio T. do N. Varella, Luis Vasquez

**Affiliations:** a Aix Marseille University, CNRS, ICR 13397 Marseille France mario.barbatti@univ-amu.fr https://www.barbatti.org; b Institut Universitaire de France 75231 Paris France; c Aix Marseille University, CINaM UMR 7325, CNRS Marseille 13288 France; d Dipartimento di Chimica e Chimica Industriale, University of Pisa 56124 Pisa Italy; e Department of Chemistry, University College London 20 Gordon St London UK; f Instituto de Física, Universidade de São Paulo São Paulo 1731 Brazil; g State Key Laboratory of Physical Chemistry of Solid Surfaces, Department of Chemistry, College of Chemistry and Chemical Engineering, and Fujian Provincial Key Laboratory of Theoretical and Computational Chemistry, Xiamen University Xiamen 361005 China; h Institute of Physics, Faculty of Physics, Astronomy, and Informatics, Nicolaus Copernicus University in Toruń ul. Grudziądzka 5 87-100 Toruń Poland; i Institute of Advanced Studies, Nicolaus Copernicus University in Toruń ul. Wileńska 4 87-100 Toruń Poland; j Aitomistic Shenzhen 518000 China; k Christian-Albrechts-University Kiel 24118 Kiel Germany; l Kiel Nano, Surface and Interface Science 24118 Kiel Germany; m Department of Chemistry, Queen Mary University of London Mile End Road London E1 4NS UK; n Scuola Superiore Meridionale Via Mezzocannone 4 I-80134 Napoli Italy; o Dipartimento di Scienze Chimiche, Università degli Studi di Napoli Federico II via Cintia 21 I-80126 Napoli Italy; p Laboratoire de Chimie et Physique Quantiques (UMR 5626), Université de Toulouse, CNRS Toulouse 31062 France; q Department of Chemistry and Biochemistry, Texas Tech University Lubbock Texas 79409-1061 USA; r Faculty of Chemistry, Nicolaus Copernicus University in Toruń ul. Gagarina 7 87-100 Toruń Poland; s J. Heyrovsky Institute of Physical Chemistry, Academy of Sciences of the Czech Republic 18223 Praha 8 Czech Republic; t Department of Chemistry, Loughborough University Loughborough LE11 3TU UK; u Université Lyon1, ENS de Lyon, CNRS, Laboratoire de Chimie, UMR 5182 Lyon France; v School of Science, Hangzhou Dianzi University Hangzhou 310018 China; w Hosei University, Computer and Information Sciences Tokyo 182-8584 Japan

## Abstract

Mixed quantum–classical dynamics (MQCD) methods are effective models for excited-state processes in quasi-classical molecular systems, in which nuclear motion is described by classical trajectories while electronic populations undergo quantum nonadiabatic transitions. This article presents Newton-X 26, a new generation of the Newton-X platform that consolidates two decades of development into a modular ecosystem for generating spectra and initial conditions, propagating dynamics, and analyzing, postprocessing, and archiving data. Newton-X 26 supports multiple MQCD strategies, including surface hopping, decoherence-corrected Ehrenfest dynamics, and *ab initio* multiple spawning, and connects to a range of electronic-structure engines through dedicated interfaces. The platform emphasizes efficient execution for large trajectory ensembles, enabling systematic convergence analyses and uncertainty estimation. Complementary tools support automated data curation, machine-learning-assisted workflows, and reproducible FAIR-oriented reporting and sharing. Taken together, Newton-X 26 provides an open-source environment for routine MQCD applications and continued method development across multiple electronic-structure levels.

## Abstract 2

Newton-X 26 unifies MQCD simulations—from surface hopping to Ehrenfest and AIMS—within a reproducible, widely interfaced, and open-source platform, facilitating method development and routine, verifiable excited-state dynamics.

## Graphical abstract

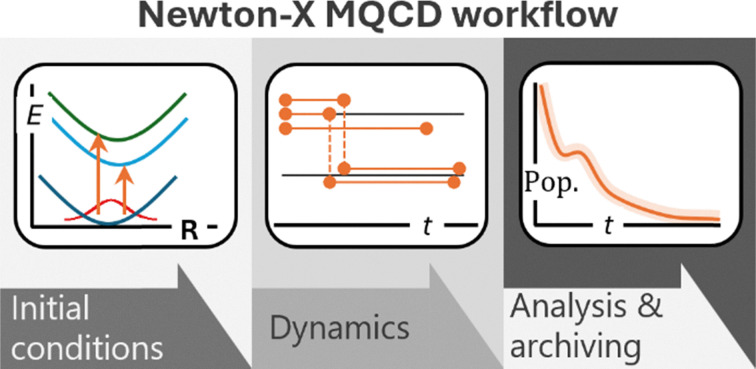

## 1 Introduction

Mixed quantum–classical dynamics (MQCD) methods, which combine classical nuclear trajectories with quantum electronic population flow, are among the most widely used approaches for nonadiabatic molecular dynamics simulations.^1,2^ Their success stems from a favorable cost–accuracy tradeoff: by exploiting the quasi-classical character of nuclear motion in many molecular systems, MQCD enables simulations of complex nonadiabatic processes at a fraction of the cost of fully quantum-mechanical treatments, while still allowing full-dimensional simulations beyond the Born–Oppenheimer approximation.

Newton-X is an open-source platform that supports integrated workflows for MQCD simulations, spanning nuclear-ensemble-based spectrum and initial-condition generation, dynamics propagation, and postprocessing. The platform supports multiple propagation paradigms—surface hopping, Ehrenfest (mean-field), and multiple spawning—driven by on-the-fly electronic-structure calculations through dedicated interfaces to third-party quantum-chemistry packages.

Newton-X has been under development for two decades. The original release, described in a 2007 paper,^3^ focused on fewest-switches surface hopping (FSSH) and a limited set of electronic-structure options. A second major paper (2014)^4^ reported substantial extensions, including decoherence corrections, time-derivative couplings, and additional quantum-chemistry interfaces. Starting in 2019, Newton-X underwent a major refactoring to improve performance and long-term maintainability, reorganizing the codebase into independent programs and enabling the incorporation of new functionalities. This modular design, first described in 2022,^5^ has now matured into Newton-X 26, which consolidates recent developments into a unified public release while maintaining compatibility with legacy inputs and established workflows.

**Fig. 1:**
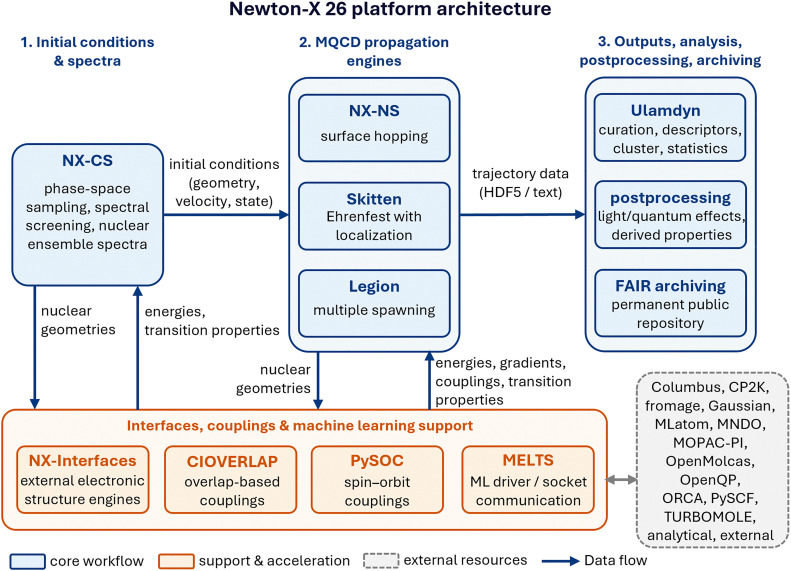
Overview of the Newton-X 26 platform. The diagram shows the separation between the core workflow modules for initial-condition generation, MQCD propagation, analysis, postprocessing, and archiving, and the support layers for electronic-structure interfaces, coupling evaluation, spin–orbit couplings, and machine-learning acceleration. Arrows indicate the main data exchanges between modules, including initial geometries, velocities, electronic states, electronic-structure quantities, trajectory data, and postprocessed observables. This layered organization allows propagation engines, coupling strategies, external electronic-structure programs, machine-learning (ML) models, and analysis tools to evolve independently while remaining part of a common Newton-X workflow. Many of these program names are acronyms, abbreviations, or wordplays. Newton-X: Newtonian dynamics near the crossing (X) seam; NX-CS: Newton-X classical series; NX-NS: Newton-X new series; Skitten: stochastic Schrödinger cats; Ulamdyn: unsupervised learning analysis of molecular dynamics; CIOVERLAP: overlaps of configuration interaction wavefunctions; PySOC: Python-based spin–orbit couplings; MELTS: MELTS efficiently learn trajectories and surfaces.

The main novelty of Newton-X 26 is the integration of complementary MQCD approaches within a common software ecosystem (Fig. 1). Surface hopping is implemented in NX-NS, now including Landau–Zener surface hopping; conventional Ehrenfest dynamics and Ehrenfest dynamics with spontaneous localization (SLED) are available through Skitten; and *ab initio* multiple spawning (AIMS) is implemented in Legion. The propagation engines are coupled to electronic-structure programs through modular Newton-X interfaces, which separate the dynamics layer from the external quantum-chemistry engines. This organization facilitates interoperability with a broader set of programs and expands the range of accessible electronic-structure descriptions and simulation setups.

The novel Newton-X 26 developments broaden the platform scope in several directions. Interfaces with CP2K and fromage enable applications to periodic systems, molecular crystals, and localized excitations in crystalline environments. The dedicated ONIOM interface with Gaussian provides a route to multilayer quantum mechanics/molecular mechanics (QM/MM) dynamics. The OpenMolcas, PySCF, and OpenQP interfaces extend the range of electronic-structure methods with balanced dynamic/static electronic correlation available for MQCD, including multireference perturbative treatments, pair-density-functional approaches, and mixed-reference spin-flip time-dependent density functional theory (TDDFT). In parallel, the linear vibronic coupling (LVC) model, used in the adiabatic representation, complements the existing analytical models in Newton-X by providing general multi-state vibronic Hamiltonians suitable for controlled tests of nonadiabatic dynamics. At the workflow level, Newton-X 26 incorporates postprocessing routes such as quantum dynamics from classical trajectories (QDCT), scripts to facilitate dataset deposition following the FAIR principles (findable, accessible, interoperable, reusable), and a more mature machine-learning (ML) acceleration layer through MELTS. Thus, Newton-X 26 is best viewed not as a replacement of the earlier Newton-X workflow philosophy, but as its consolidation into a broader, multi-method, and more interoperable MQCD platform.

Beyond the specific programs distributed with Newton-X 26, the platform also illustrates several design principles that are transferable to other computational-chemistry software ecosystems. First, propagation algorithms, electronic-structure interfaces, coupling evaluators, machine-learning drivers, and analysis tools are separated into distinct layers that communicate through well-defined data exchanges rather than through monolithic code dependencies. Second, the same ecosystem accommodates different MQCD paradigms—surface hopping, Ehrenfest/SLED, and AIMS—so that methodological diversity is handled within a common software architecture rather than through isolated single-method codes. Third, trajectory outputs are treated as reusable scientific data, with postprocessing, compact storage, and FAIR-oriented deposition considered part of the simulation workflow rather than optional afterthoughts. These choices are implemented here in Newton-X 26, but the underlying strategy—modular propagation engines connected to interoperable electronic-structure, ML, and data-analysis layers—is broadly relevant for the sustainable development of molecular-simulation software.

**Fig. 2:**
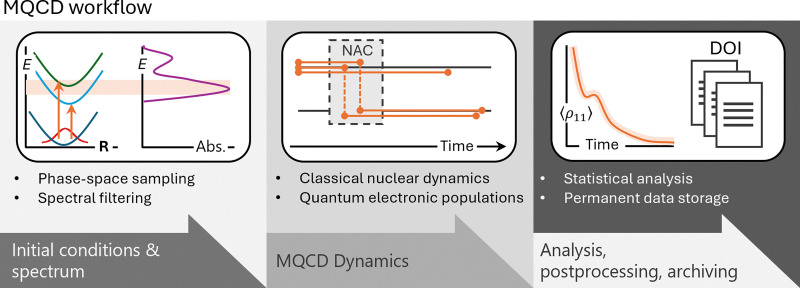
MQCD workflow in Newton-X 26. Simulations proceed from initial-condition generation and spectrum preparation (phase-space sampling and spectral filtering), to mixed quantum–classical dynamics where classical nuclei are propagated alongside quantum electronic population transfers, and finally to ensemble-based analysis, postprocessing, and archiving of results with permanent identifiers.

In this paper, we present the Newton-X 26 platform following the three basic steps of an MQCD workflow (Fig. 2): (1) spectra and initial conditions; (2) dynamics simulations; and (3) analysis, postprocessing, and archiving. For each step, we outline the relevant concepts and describe how they are implemented in Newton-X.

## 2 Spectrum and initial conditions

MQCD simulations rely on ensembles of classical trajectories. A crucial first step is therefore the generation of initial conditions, which specify the initial nuclear geometries and velocities, as well as the initial electronic states from which the trajectories are launched. In practice, this preparation can be viewed as two workflow components: (i) sampling the nuclear phase space of a chosen source state, and (ii) applying a spectral screening to select which sampled geometries will initiate trajectories on a chosen target state manifold (Fig. 2-left).

In the most common MQCD scenario, a system prepared in a source state—typically the electronic ground state—is promoted to one or more target excited states chosen to initiate the nonadiabatic dynamics. Nuclear geometries and velocities can be obtained from a Wigner distribution within the harmonic approximation1
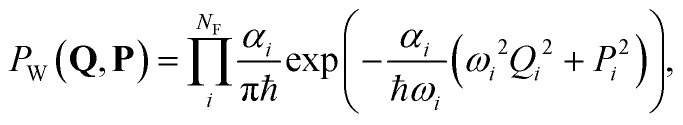
where *α*_*i*_ = tanh(*ħω*_*i*_/(2*k*_B_*T*)) and *N*_F_ is the number of degrees of freedom (*N*_F_ = 3*N*_at_ − 6 for a molecule with *N*_at_ atoms). In these equations, *Q*_*i*_ = *μ*_*i*_^1/2^*q̃*_*i*_ and *P*_*i*_ = *μ*_*i*_^−1/2^*p̃*_*i*_ are the mass-scaled coordinate and momentum for each normal mode *i* with coordinate *q̃*_*i*_, momentum *p̃*_*i*_, reduced mass *μ*_*i*_, and angular frequency *ω*_*i*_. Alternatively, sampling coordinates and momentum from a previous dynamics run or from other sampling schemes representing the phase-space distribution of the source state is also possible.^6–8^

Once a set of candidate geometries and velocities is available, quantum chemical calculations can be run for each geometry **R**_*n*_ to collect excitation energies Δ*E*_1*L*_(**R**_*n*_) and oscillator strengths *f*_1*L*_(**R**_*n*_) between the ground state (denoted 1) and the excited state (*L*). Then, spectral screening selects the subset used for trajectory initiation according to additional criteria, such as transition probabilities between source and target states or—in the case of incoherent excitation^9^—the spectral radiance of the incident light. A common option for such a screening is to accept an initial condition starting from state *L* if the double criteria2
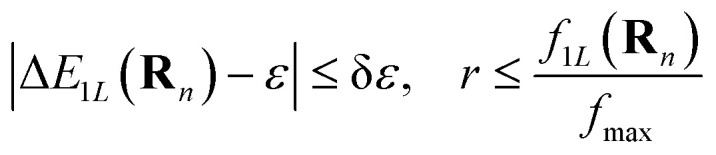
is satisfied. Here, *r* is a uniform random number in the [0,1] interval, *f*_max_ is the maximum oscillator strength in the ensemble, and *ε* ± δ*ε* is the energy window. This procedure yields a set of initial conditions starting from multiple adiabatic states, biased toward bright states.

The same electronic-structure information used for screening also provides access to the system's transition spectrum. For example, when the source state is the ground state and the target states are electronically excited, the distribution of vertical excitation energies weighted by oscillator strengths yields a nuclear-ensemble approximation (NEA) to the steady-state absorption cross section^10^3
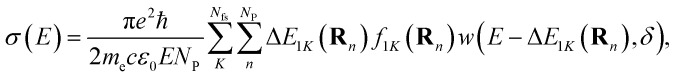
where *E* is the photon energy, *e* is the elementary charge, *m*_e_ is the electron mass, *ε*_0_ is the vacuum permittivity, and *ħ* is the reduced Planck's constant. *N*_P_ is the number of sampled geometries **R**_*n*_, *N*_fs_ is the number of final electronic states included in the spectrum and *w*(*E* − Δ*E*_1*K*_(**R**_*n*_), *δ*) is a normalized line-shape function with a broadening parameter *δ*. Examples of NEA phosphorescence and fluorescence spectra are discussed in ref. 11 and 12.

Within the Newton-X 26 platform, the NX-CS program implements this initial condition and NEA workflow, combining multiple phase-space generation algorithms with flexible spectral screening through user-defined excitation windows. Trajectories can thereby be assigned stochastically to one or more adiabatic initial states according to transition probabilities. The selected ensemble then serves as input for the Newton-X propagation programs (NX-NS, Legion, Skitten), providing a consistent bridge between spectral preparation and MQCD dynamics.

## 3 Dynamics simulations

Dynamics propagation is the core step of an MQCD workflow (Fig. 2-middle), and usually dominates the computational cost. This is also where the central modeling decision is made: selecting an MQCD strategy that matches the target observables. That choice is constrained by the feasible electronic-structure level^13^ and by practical model ingredients such as environmental effects,^14^ geometric restrictions, and treatments for zero-point energy leakage.^15^ The following subsections summarize the MQCD propagation methods available in Newton-X (Table 1) and the practical choices that control their cost, robustness, and accuracy.

**Table 1:** MQCD algorithms available in Newton-X 26, together with the quantities used to drive electronic transitions or population transfer, and the available decoherence/localization^16,17^ treatments. Nonadiabatic information can be provided as nonadiabatic coupling vectors (NACVs) or as wavefunction overlaps (OVL). It may also be evaluated through the time-dependent Baeck–An (TD-BA) approximation.^18^ In surface hopping, decoherence corrections include the simplified decay of mixing (SDM)^19^ and an overlap-driven decoherence scheme (OD).^20^ Ehrenfest dynamics is available in pure mean-field form, while the Ehrenfest dynamics with spontaneous localization (SLED)^17^ extension introduces decoherence through spontaneous localization

MQCD algorithm	Nonadiabatic coupling	Decoherence correction
Fewest switches surface hopping (FSSH)^21^	NACV, TD-BA, OVL	SDM, OD
Complex-surface FSSH (CS-FSSH)^22^	NACV	SDM
Local-diabatization surface hopping (LDSH)^23^	OVL	SDM, OD
Landau–Zener surface hopping (LZSH)^24^	Curvature-based	—
Ehrenfest (mean-field) dynamics^25^	NACV	SLED
*Ab initio* multiple spawning (AIMS)^26^	NACV, TD-BA, OVL	—

Surface hopping, discussed in Section 3.1, remains the most mature and widely developed propagation framework in Newton-X. It is the approach of choice for routine simulations aimed at tracking nonadiabatic events and estimating population-transfer time constants. Newton-X 26 also includes complementary approaches based on Ehrenfest dynamics with spontaneous localization (SLED; Section 3.2) and *ab initio* multiple spawning (AIMS; Section 3.3). SLED is aimed at cases where the mean-field picture is attractive, but where fully coherent Ehrenfest evolution would give an incomplete description. At this stage, however, SLED should be regarded as a developing capability within Newton-X 26, less mature and less widely benchmarked than surface hopping. AIMS provides a high-level trajectory-guided wavepacket reference for problems in which nonadiabatic branching and electronic coherence are central.

### 3.1 Surface hopping

**Fig. 3:**
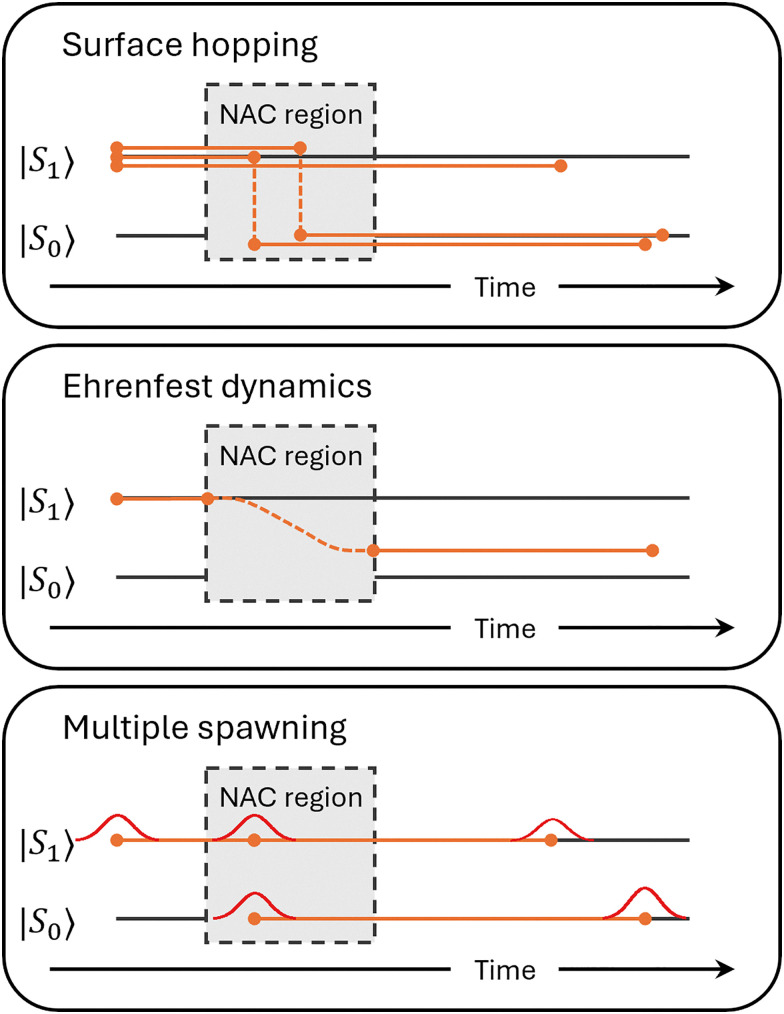
Schematic comparison of MQCD propagation strategies implemented in Newton-X. In surface hopping (top), an ensemble of independent classical nuclear trajectories evolves on adiabatic electronic states and undergoes stochastic transitions in regions of strong nonadiabatic coupling (NAC). In Ehrenfest dynamics (middle), a single trajectory is propagated under a mean-field force, with electronic populations transferring smoothly through the NAC region. Trajectory ensembles are built considering different initial conditions. In multiple spawning (bottom), the nuclear wavefunction is represented by trajectory-guided basis functions that can branch (spawn) onto different electronic states during the nonadiabatic event, yielding a superposition of wavepacket components after the coupling region.

Surface hopping propagates classical nuclear trajectories on a single adiabatic electronic state at a time, while allowing stochastic transitions (hoppings) between states due to nonadiabatic couplings (Fig. 3-top). Ensemble averaging over many trajectories provides an approximate description of quantum wavepacket evolution. Because trajectories are independent, surface hopping is computationally efficient and well-tailored for on-the-fly computation algorithms.^27^ After a successful hopping, nuclear velocities are adjusted to enforce total energy conservation; if this cannot be achieved, the hop is rejected (a frustrated hopping), depending on the chosen velocity-adjustment scheme.^28^

In Newton-X, surface hopping is implemented in the NX-NS program. NX-NS supports both instantaneous and global hopping schemes. In instantaneous schemes, hopping probabilities are evaluated at each time step. The probabilities are computed from the propagated electronic coefficients, obtained by integrating the electronic time-dependent Schrödinger equation (TDSE)4
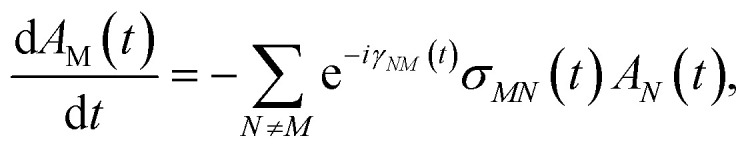
along the classical nuclear trajectory. In this expression, which assumes adiabatic representation, *A*_*M*_ are the electronic coefficients determining the quantum state5
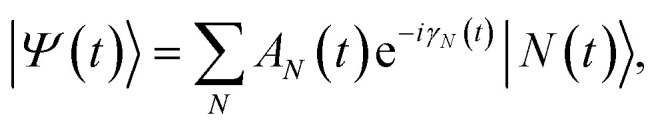
and the time-derivative couplings (TDC) *σ*_*MN*_ are6
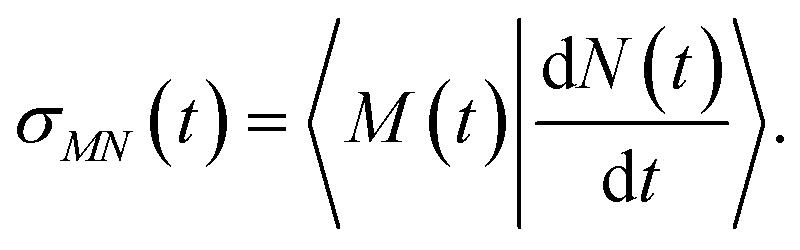


The time-dependence of the adiabatic states |*M*(*t*)〉 arises from their parametric dependence on the nuclear geometry **R**(*t*), which is obtained through the classical equations for each nucleus *α* with mass *M*_*α*_,7
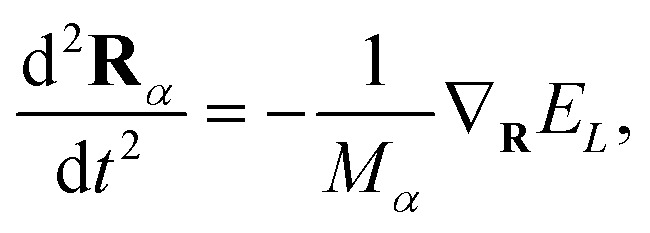
for a system in state *L* with potential energy *E*_*L*_. In eqn (5), the phase is 
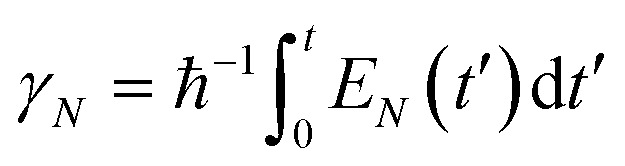
 and the phase difference in eqn (4) is *γ*_*MN*_ = *γ*_*M*_ − *γ*_*N*_. Both FSSH^21^ and local-diabatization surface hopping (LDSH)^23,29^ solve these equations, although they integrate eqn (4) in completely different ways. In FSSH, the result yields the *L* → *M* hopping probability8
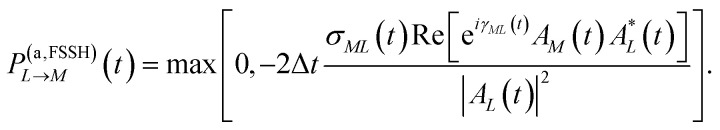
In LDSH, the probability is given by eqn (19) of ref. 23.

The integration of the electronic TDSE (eqn (4)) within the independent-trajectory approximation can misrepresent electronic coherence—quantified by the off-diagonal elements of the electronic density matrix constructed from the coefficients—motivating the use of decoherence corrections.^16,19,30,31^ The main decoherence correction used in NX-NS is the simplified decay of mixing (SDM).^19^ In SDM, after propagation over a nuclear time step Δ*t*, the coefficients of the inactive states are damped according to9
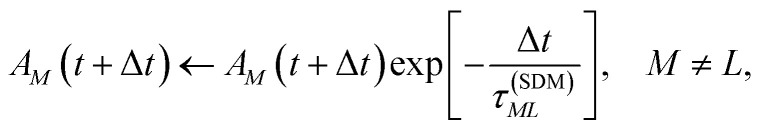
where *L* is the active state and10
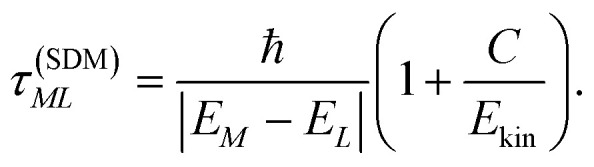
Here, *E*_kin_ is the nuclear kinetic energy, and *C* is an empirical parameter. The coefficient of the active state is then rescaled to preserve normalization,11
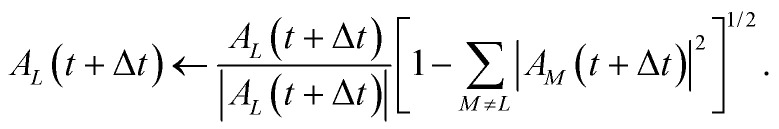
Thus, SDM damps electronic coherence between the active and inactive states while leaving population transfer governed by the underlying hopping algorithm.

NX-NS also supports CS-FSSH, a FSSH propagation method for complex-valued potential energy surfaces (PESs) arising from non-Hermitian Hamiltonians, allowing the simulation of dissipative processes.^22^ The various approaches for computing the nonadiabatic couplings required for the TDSE integration are discussed in Section 3.6.

Global (or asymptotic) hopping schemes are based on asymptotic transition probabilities, such as Landau–Zener surface hopping (LZSH) within the Belyaev–Lebedev adiabatic formulation^32–34^12
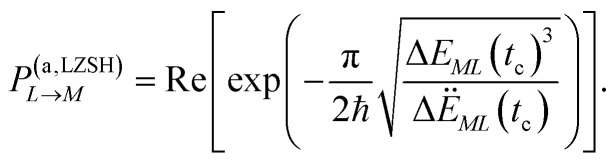
In this expression, the hopping probability *L* → *M* depends on the energy gap Δ*E*_*ML*_ = *E*_*M*_ − *E*_*L*_ and its second derivative in time, Δ*Ë*_*ML*_ at the time *t*_c_ of the minimum gap.

Conceptually, these probabilities can be viewed as the asymptotic outcome of integrating a local two-state electronic TDSE in the vicinity of a coupling (avoided-crossing) region;^35^ in practice, one uses the resulting closed-form Landau–Zener-type expression rather than explicitly propagating electronic coefficients. In the adiabatic representation, the transition probability depends on the minimum energy gap and its second-order time derivatives.^24^ In NX-NS, this asymptotic evaluation is performed using a three-point criterion,^34^ whereby the hopping is evaluated at time *t* when the energy gap exhibits a local minimum relative to *t* − Δ*t* and *t* + Δ*t*. The Landau–Zener formula itself does not assume decoherence, as it is derived from asymptotic coherent scattering amplitudes in a semiclassical two-state model.^35^ In LZSH, however, that probability is used to assign the trajectory to one adiabatic branch after the crossing, so coherence between the alternative outgoing pathways is no longer propagated.^16^ LZSH therefore incorporates decoherence only in a simplified branching picture.

Either with instantaneous or global probabilities, a tentative hopping *L* → *J*_*q*_ is selected when13
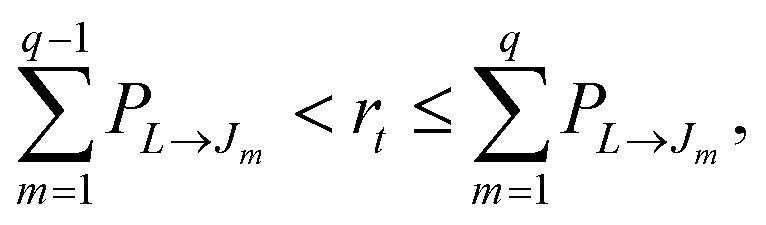
where {*J*_*m*_} is an ordered list of states excluding *L* and *r*_*t*_ is a uniform random number drawn from the [0,1] interval. The tentative hopping is accepted if the kinetic energy reservoir along the velocity-rescaling direction **u**,^28^14
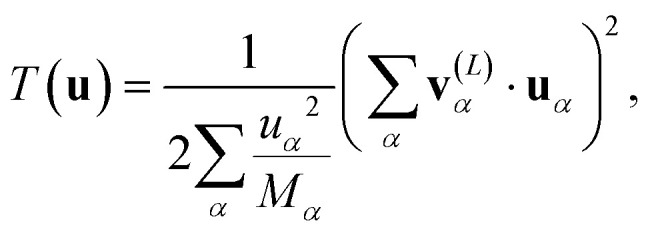
is sufficient to compensate for the potential energy gap, *i.e.*,

15 Δ*E*_*J*_*q*_*L*_ = *E*_*J*_*q*__ − *E*_*L*_ ≤ *T*(**u**).

Otherwise, the hopping is frustrated.

The NACV is the most physically motivated choice of **u**.^36,37^ If NACV is not available, the gradient difference direction is the next best option.^28^ In this case, NX-NS can compute the necessary gradients only at the hopping evaluation to minimize the costs. The nuclear momentum **p** can also be chosen as the direction **u**. However, it may lead to an overestimation of the kinetic energy reservoir, thereby reducing the number of frustrated upward hops and affecting the detailed hopping balance. This problem can be alleviated using the reduced kinetic energy reservoir^28^*T*_red_(**p**) = *T*(**p**)/*N*_F_, where *N*_F_ is the number of vibrational degrees of freedom. After an accepted hopping, nuclear velocities should be rescaled in the direction of **u** to ensure total energy conservation.

NX-NS supports the standard separation of nuclear and electronic integration time scales. Nuclear propagation can be performed with a larger time step. In turn, the electronic equations are integrated with a smaller time step, using interpolation to obtain the required electronic quantities between electronic-structure evaluations. This multiple-time-step strategy is important for enabling surface-hopping simulations with expensive electronic-structure methods (*e.g.*, MRCI and CASPT2), for which on-the-fly calculations at very small nuclear time steps would be prohibitive.

Finally, NX-NS includes the Hessian-free local-pair zero-point-energy leakage correction (LP-ZPE),^15^ tailored for on-the-fly dynamics, which reduces unphysical energy flow from high-frequency modes while minimally perturbing the trajectory propagation.

### 3.2 Ehrenfest dynamics

In Ehrenfest dynamics (Fig. 3-middle), trajectories are propagated on a weighted average of PESs, with the weights determined by the electronic-state coefficients obtained from the electronic TDSE along the classical nuclear coordinates.^17,25,38,39^ As with surface hopping, Ehrenfest is an independent-trajectory approach and, in its standard form, lacks electronic decoherence. In Newton-X, this deficiency can be alleviated using Ehrenfest dynamics with spontaneous localization (SLED), available in the Skitten program.^17^

SLED adds a dissipative stochastic term to the electronic TDSE, eqn (4), in the form of the Gisin–Percival quantum-state diffusion equation,^40^16
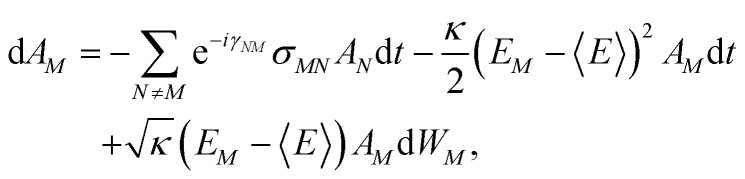
where the expected energy value is17
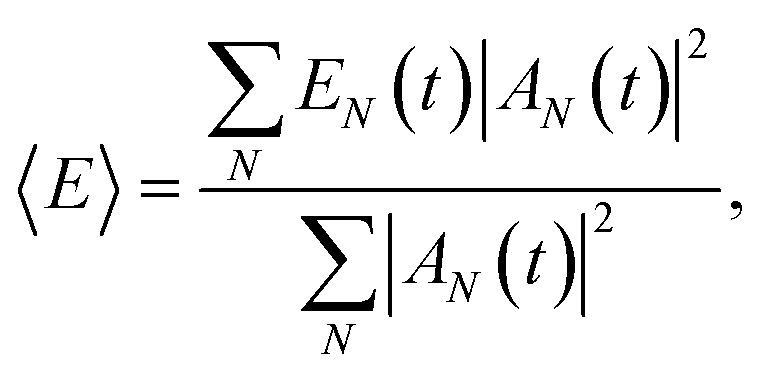
d*W*_*M*_ is a complex Wiener process, *κ* is a real and positive localization kernel encoding the electron-nuclear coupling strength, and **A** must be normalized at each time step. The nuclear coordinates are propagated in a mean field through18
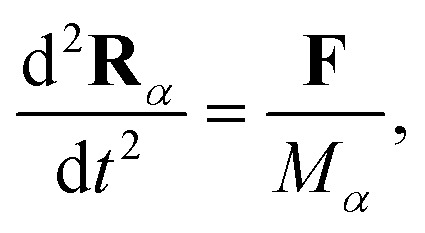
where19
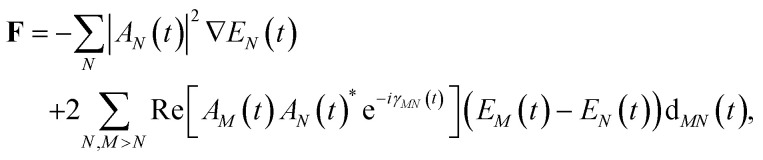
and **d**_*MN*_ = 〈*M*|∇_**R**_*N*〉 is the nonadiabatic coupling vector (NACV). This approach corresponds to Ehrenfest (mean-field) dynamics if *κ* = 0 in eqn (16).

With these equations, the electronic state evolves stochastically in the adiabatic energy basis, producing trajectory-level localization and continuous decoherence. At the ensemble level, these stochastic trajectories correspond to a Lindblad-type propagation of the reduced electronic density matrix, linking MQCD to an open-quantum-system description.^17,41^

Total energy conservation in SLED is enforced by velocity adjustments analogous to those used in surface hopping but applied at each timestep. Ehrenfest propagation requires the electronic amplitudes at each time step to compute the mean-field force on the nuclei; therefore, electronic and nuclear equations must be advanced together and cannot be decoupled as in surface hopping. Thus, Skitten propagates Ehrenfest dynamics exclusively using analytical models or machine-learning potentials, which are fast enough for the timesteps required by the coupled quantum–classical integration.

The stochastic localization term in SLED introduces trajectory-level decoherence while retaining continuous feedback of the electronic amplitudes on the nuclear force, offering an alternative to the more discontinuous electronic–nuclear feedback of surface hopping. More broadly, because the stochastic trajectories can be viewed as an unraveling of a Lindblad-type master equation, SLED opens Newton-X to open-quantum-system descriptions of dissipative and decohering excited-state dynamics.

### 3.3 *Ab initio* multiple spawning

*Ab initio* multiple spawning (AIMS) propagates classical trajectories associated with frozen Gaussian basis functions and, under prescribed criteria, spawns new trajectory basis functions (TBFs), for instance, in regions of strong nonadiabatic coupling (Fig. 3-bottom).^26,42^ Each individual trajectory is propagated using the classical equations of motion, eqn (7). The classical nuclear position **R̄** and momentum **P̄** are used to define the frozen Gaussian for that trajectory,20
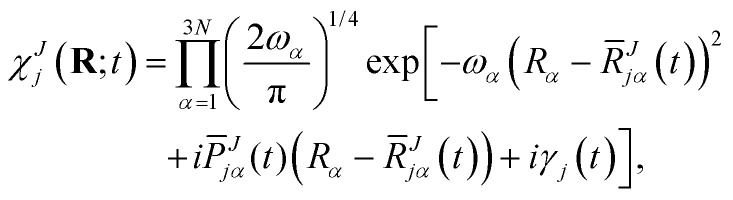
where *α* runs over the nuclear coordinates, *ω*_*α*_ is the width of the Gaussian function for each atom type, and *γ* is the phase factor, which can also be propagated as21
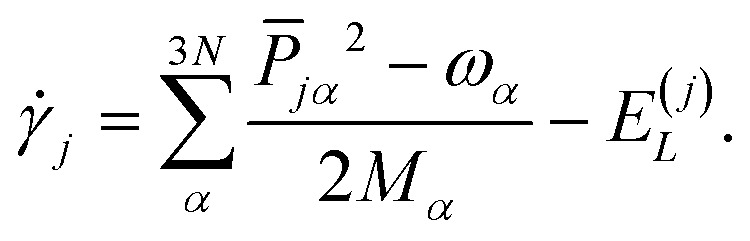
Here, *E*^(*j*)^_*L*_ is the electronic energy of the current state (*L*) of trajectory *j*.

Multiple trajectories are propagated simultaneously, and the molecular wavepacket is formed by their linear combination, using the Born–Huang representation,^26^22
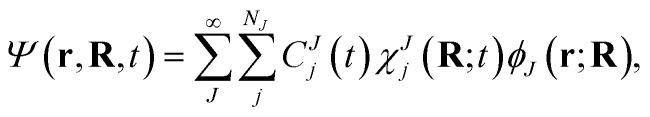
with *ϕ*_*J*_ being the electronic wavefunction of state *J*. The quantum description of the system comes from the propagation of the coefficients **C**. Inserting eqn (22) into the TDSE, one obtains the equation of motion for the coefficients,

23 **Ċ** = −*i***S**^−1^(**T** + **V** − **τ** − *i***Ṡ**)**C**.

The matrix elements for the kinetic **T**, potential **V**, coupling **τ**, and overlap **S** matrices are defined in eqn (6) of ref. 43.

To describe the non-classical region, a spawning algorithm can deterministically generate new TBFs on the same or on different electronic states of existing trajectories. While each trajectory is propagated classically, parent and daughter basis functions remain quantum-mechanically coupled through their mutual overlap, since the nuclear wavefunction is represented as a superposition of frozen Gaussians centered at the classical nuclear coordinates. This coupling between subsets of trajectories already provides a more explicit treatment of electronic coherence than obtained with surface hopping or Ehrenfest dynamics. Nevertheless, the potentially high spawning rate can make AIMS computationally demanding, motivating algorithms that prune weakly coupled basis functions and manage the size of coupled subsets.^44^

The Legion program^43^ implements AIMS within Newton-X 26, including classical trajectory propagation, spawning, pruning, coefficient propagation, coupling evaluation, and Gaussian-basis parametrization. It can use explicit NACVs when available, but it also supports TDC-based treatments obtained either from wavefunction overlaps or from the time-dependent Baeck–An approximation (see Section 3.6). By reducing the dependence on explicit NACVs, these options broaden the range of electronic-structure methods that can be coupled to AIMS within Newton-X (Section 3.4). In addition, Legion provides element-specific parameters for frozen Gaussian basis functions across the periodic table.

In Legion, spawning is controlled by monitoring either the NACV norm or the corresponding TDC along each parent trajectory. When the selected coupling measure exceeds a user-defined threshold, the parent trajectory is followed through the coupling region, and a child TBF is created at the maximum-coupling point on the coupled electronic state. The child initially inherits the parent geometry, while its momentum is adjusted to conserve total energy, typically along either the NACV or the nuclear-momentum direction; it is then backpropagated to the beginning of the coupling region. This construction allows population transfer to emerge from the quantum propagation of the TBF coefficients rather than from a stochastic hopping rule. To control the growth of the Gaussian basis, Legion also supports pruning strategies that remove weakly coupled, weakly overlapping, and negligibly populated TBFs only after satisfying the pruning criteria over a prescribed time window; the wavepacket is then projected onto the reduced basis. Expensive electronic-structure calculations at TBF centroids can be avoided through bra–ket averaged Taylor-type approximations, including BAT and related variants.

In this way, Legion extends Newton-X beyond independent-trajectory MQCD while preserving the workflow logic of the other propagation engines. Its modular structure makes AIMS a flexible implementation layer, allowing spawning criteria, pruning strategies, coupling evaluation, centroid approximations, and Gaussian-basis parameters to be modified or replaced. It also facilitates extensions to related trajectory-guided quantum wavepacket methods, such as the quantum dynamics from classical trajectories (QDCT) approach,^45^ discussed in Section 4.2.

### 3.4 Electronic-structure methods and interfaces

MQCD requires potential energy gradients to propagate classical nuclear trajectories, and potential energies and nonadiabatic couplings to drive electronic-state transitions. These quantities are typically obtained from electronic-structure calculations at each classical nuclear geometry. Because couplings can also be estimated by alternative schemes, they are discussed separately in Section 3.6. Since Newton-X separates these propagation requirements from the specific electronic-structure engine, the same MQCD framework can be coupled to a broad range of quantum-chemistry methods, from *ab initio* multireference configuration interaction (MRCI) to time-dependent density functional theory (TDDFT) (Table 2). The most appropriate level of theory depends on the target system and observables, and must balance accuracy against computational affordability.^13^

**Table 2:** Potential-energy-surface (PES) methods available for MQCD simulations in the Newton-X 26 platform, along with the corresponding quantum-chemistry packages that provide them *via* the NX-interfaces program. The quantum chemistry programs are not distributed with Newton-X and must be obtained separately. When available, Newton-X uses nonadiabatic coupling vectors (NACVs); otherwise, it estimates couplings from wavefunction overlaps (OVLs), which can be used for time-derivative couplings (TDCs) or local diabatization. TDCs can also be estimated *via* the time-dependent Baeck–An (TD-BA) approximation

Quantum chemistry packages	Electronic-structure method^a^	Nonadiabatic coupling
Built-in analytical models	1D collection;^21,46–48^ SBH^49^	NACV, TD-BA
	CS-1D models^22^	NACV
	LVC^50^	OVL, TD-BA
Columbus^51^	MRCI;^52^ MCSCF^53^	NACV, OVL, TD-BA
TURBOMOLE^54^	ADC(2);^55^ CC2;^56^ TDDFT;^57^ TDA^57^	OVL, TD-BA
Gaussian^58^	TDDFT; TDA	OVL, TD-BA
ORCA^59^	TDDFT; TDA	OVL, TD-BA
OpenMolcas^60^	CASSCF;^61^ CASPT2;^62^ CMS-PDFT^63^	NACV, TD-BA
OpenQP^64^	MRSF-TDDFT^65^	TD-BA
MOPAC-PI^66^	FOMO-CI^23^	NACV, OVL, TD-BA
CP2K^84^	TDA;^67^ sTDA;^67^ sTDA-GFN1-xTB,^68^ SF-TDA^69^	OVL, TD-BA
PySCF^70^	#-PDFT^63^	NACV, TD-BA
Tinker^71^–MNDO^72^	ODM*x*/MRCI^73^	NACV, TD-BA
fromage^74^	All methods available in fromage	TD-BA
MLatom^75^	ML potentials; AIQM1/CI^76^	TD-BA

a 1D collection: one-dimensional model-trajectory test set (Tully models and other one-dimensional nonadiabatic potential energy profiles); CS-1D models: collection of complex-valued one-dimensional nonadiabatic potential energy profiles; SBH: spin–boson Hamiltonian; LVC: linear vibronic coupling; MRCI: multireference configuration interaction; MCSCF: multiconfigurational self-consistent field; ADC(2): second-order algebraic-diagrammatic construction; CC2: second-order approximate coupled cluster; TDDFT: time-dependent density functional theory; TDA: Tamm–Dancoff approximation; CASSCF: complete active space self-consistent field; CASPT2: complete active space second-order perturbation theory; CMS-PDFT: compressed multistate pair-density functional theory; MRSF-TDDFT: mixed-reference spin-flip TDDFT; FOMO-CI: floating occupation molecular orbital configuration interaction (with semiempirical Hamiltonians); sTDA: simplified TDA; GFN1-xTB: geometry, frequency, noncovalent—extended tight-binding version 1 model; SF-TDA: spin-flip TDA; #-PDFT: pair-density functional theory variants, where # specifies the particular flavor used; ODMx: orthogonalization- and dispersion-corrected semiempirical method (*x* denotes the specific parametrization, *e.g.*, ODM2/ODM3); ML: machine learning; AIQM1/CI: configuration interactions based on artificial-intelligence quantum-mechanics method 1.

Except for analytical model potentials implemented internally, all other electronic-structure methods rely on interfaces to third-party programs distributed separately. The quantities that must be returned by the electronic-structure engine depend on the selected MQCD method, coupling strategy, and velocity-adjustment scheme; the main cases are summarized in Table 3. This communication is handled by NX-interfaces, a set of programs that dynamically generate input files, execute an external code, and parse the required quantities from the external code's output. NX-interfaces is developed as standalone tools that can be called from external workflows, and they can also be compiled into Fortran libraries. Newton-X also provides a general interface that can wrap arbitrary electronic-structure codes, provided they return the quantities required by the chosen MQCD method—in particular, state energies, state gradients, nonadiabatic coupling vectors, or wavefunction-overlap information—in a Newton-X-readable exchange format. The currently supported electronic-structure methods and associated programs are summarized in Table 2.

**Table 3:** Electronic-structure quantities requested by Newton-X and their main roles in the MQCD workflow. Not all quantities are required for every simulation. The requested data depend on the selected propagation method, coupling strategy, velocity-adjustment scheme, and whether the calculation is used for initial-condition generation, dynamics, or postprocessing. Wavefunction-overlap ingredients may include atomic-orbital overlaps between consecutive time steps together with molecular-orbital, CI, or linear-response coefficients, depending on the electronic-structure method

Electronic-structure quantity	Main use in Newton-X
Adiabatic energies *E*_*M*_(**R**)	Initial-state assignment, spectral screening, and nuclear-ensemble spectra; electronic TDSE integration; phase accumulation; monitoring of dynamics; hopping-probability evaluation; Ehrenfest/SLED mean-field force; SLED localization; AIMS Hamiltonian matrix elements
Gradient of the active/current state ∇*E*_*L*_(**R**)	Classical nuclear propagation in surface hopping and AIMS; mean-field force in Ehrenfest/SLED
Gradient of additional states ∇*E*_*M*_(**R**)	Mean-field force in Ehrenfest/SLED; velocity adjustment after hops when gradient-difference rescaling is used
Nonadiabatic coupling vector **d**_*MN*_(**R**)	Electronic TDSE integration; NACV-based velocity rescaling; AIMS coupling matrix elements; Ehrenfest/SLED mean-field force
Wavefunction-overlap ingredients between consecutive time steps	Overlap-based TDCs in FSSH and transformation matrix in LDSH; overlap-based coupling evaluation in AIMS
Oscillator strengths *f*_*MN*_(**R**)	Initial-state assignment, spectral screening, and nuclear-ensemble spectra; monitoring of dynamics

In practice, these interface files contain the stepwise electronic-structure data needed for propagation, while the dynamics outputs may additionally record quantities such as geometries, velocities, active states, populations, and nonadiabatic events. In NX-NS, user-defined external scripts can also be executed during propagation, enabling on-the-fly monitoring of arbitrary quantities or custom analysis and handling of selected outputs. The amount of data written during the simulation can be controlled through output options, allowing users to retain only the information needed for a given workflow and to limit storage overhead in large trajectory ensembles. For subsequent consolidation and postprocessing, these data can also be organized in H5MD,^77^ an HDF5-based format that offers compact storage, efficient read/write operations, and straightforward access from common analysis environments.

Newton-X can also handle environmental, hybrid, and extended-system setups, as discussed in Section 3.5. In addition, it supports MQCD dynamics with machine-learning potentials, as described in Section 3.7.

### 3.5 Environmental, hybrid, and extended-system setups

Environmental effects can be incorporated in excited-state dynamics at different levels of approximation, including atomistic models, continuum solvation approaches,^78^ and multiscale strategies in which different regions of the system are treated at different levels of theory, as in QM/MM schemes.^79^ The most appropriate choice depends on how explicitly the environment must be described and at what computational cost.^80^ In Newton-X, these alternatives can be incorporated largely independently of the specific environment model, provided that each time step supplies the quantities required by the chosen MQCD method; the main supported strategies are summarized in Table 4.

**Table 4:** Simulation setups available in Newton-X beyond standard isolated-molecule on-the-fly dynamics, including hybrid QM/MM embedding, fragment and excitonic models, periodic or crystalline environments, and open-system and dissipative dynamics. For each setup, the table indicates the corresponding approach, the underlying electronic Hamiltonian or electronic-structure level, and the quantum chemistry package used

Simulation setup	Approach^a^	Underlying electronic Hamiltonian	Quantum chemistry packages
Hybrid setups	ONIOM(QM:M1[:M2])^81,82^	QM: TDDFT; TDA M1/M2: DFT; semiempirical; MM; continuum methods	Gaussian
	QM/MM^14^	OD*Mx*/MRCI	Tinker–MNDO
	QM/polarizable MM^14^	TDDFT; TDA	Gaussian–Tinker
Fragment and excitonic models	EXASH^83^	FOMO-CI	MOPAC-PI–Tinker
	EXASH	TDDFT; TDA	Gaussian–Tinker
Periodic and crystalline systems	GPW/GAPW basis^84^	TDA; sTDA; SF-TDA	CP2K
	ONIOM(QM:QM′)	QM: methods available through fromage QM′: xTB; DFTB	fromage^74^
Open-system and dissipative dynamics	CS-FSSH^22^	Complex-valued PES	Columbus; Newton-X built-in approaches
	SLED^17^	Analytical models	Newton-X built-in approaches

a CS-FSSH: complex-surface fewest switches surface hopping; SLED: Ehrenfest dynamics with spontaneous localization; ONIOM: our own *n*-layered integrated molecular orbital and molecular mechanics; QM/MM: quantum mechanics/molecular mechanics; EXASH: exciton approach for surface hopping; GPW/GAPW: Gaussian and plane waves/Gaussian and augmented plane waves.

Some of the environmental and extended-system capabilities in Table 4 are described in dedicated publications. In contrast, others are interface-level implementations whose methodological ingredients are inherited from the underlying electronic-structure or embedding programs. For this reason, this section distinguishes between the Newton-X propagation requirements, the external electronic-structure/environment model, and the current practical limitations of each setup.

In practice, Newton-X requires only the quantities needed by the selected MQCD approach, such as electronic-state energies, nuclear gradients, and, when necessary, nonadiabatic couplings or overlap-based quantities (Table 3). In hybrid approaches, it may also require defining the region in which nonadiabatic processes are treated explicitly. Once this information is available, the dynamics can be propagated in the usual way, allowing Newton-X to exploit the broad range of environment models available in the electronic-structure programs to which it is interfaced.

While this general interface-based design already provides access to many environmental treatments implemented in third-party programs, some setups have also been incorporated *via* dedicated Newton-X interfaces that provide tighter integration for specific hybrid strategies. A notable dedicated implementation is the subtractive multilayer ONIOM approach in the Newton-X/Gaussian interface.^81,82^ It supports two- and three-layer setups, with the nonadiabatic region restricted to the innermost QM layer. In contrast, the outer layers are treated within the ONIOM framework available in Gaussian (including both QM and MM methods). Gaussian assembles the extrapolated energies and forces and passes them to NX-NS for propagation. The implementation can handle boundaries crossing covalent bonds, is compatible with implicit solvation models, and also supports electrostatic embedding when environmental polarization should act directly on the electronic structure of the active region. In addition, Gaussian's external keyword allows the middle or outer ONIOM layers to be supplied by external programs through user-provided scripts, extending the range of hybrid setups accessible in practice.^81,82^

Another dedicated hybrid-method implementation is the Tinker–MNDO interface, which was specifically designed for additive QM/MM excited-state dynamics. In this scheme, Tinker calls MNDO at each step and transfers the resulting quantities to Newton-X. The interface relies on MNDO for electrostatic embedding, while Tinker provides the molecular-mechanics terms needed to describe QM/MM nonbonded interactions and the energy contributions associated with link atoms. In practical atomistic QM/MM applications using explicit nonadiabatic couplings, these are commonly evaluated only for the QM region, since including MM contributions would be much more expensive and is usually expected to have little effect when the QM/MM partition is physically well chosen. At present, only nonpolarizable force fields are supported by this interface.

Polarizable environments, such as those described by the AMOEBA force field,^85^ pose additional theoretical and practical challenges for nonadiabatic dynamics.^86^ Newton-X has also been interfaced with a development version of Gaussian to enable TDDFT/AMOEBA surface-hopping simulations, although this implementation is not yet publicly available.^14^

For multichromophoric systems, interfaces with MOPAC-PI and Gaussian enable the exciton approach for surface hopping (EXASH).^83^ In this fragment-based QM/MM strategy, each chromophore is treated quantum mechanically in the molecular-mechanical environment generated by the others, and the fragment-level results are assembled into the excitonic electronic-structure quantities used to propagate the dynamics.^83^

Newton-X also supports condensed-phase and solid-state simulations through the CP2K interface, which enables surface-hopping dynamics under periodic boundary conditions for systems such as molecular crystals, surfaces, porous materials, and liquids.^87^ In this setup, excited-state dynamics can be driven by TDA-based electronic structure from CP2K and combined with the overlap and TD-BA coupling strategies available in Newton-X (Section 3.6). The interface builds on CP2K's Gaussian-and-plane-waves (GPW) framework, which combines a plane-wave representation of the density with localized Gaussian orbitals.^84^ This dual representation benefits from the intrinsic periodicity of plane waves while retaining the locality of Gaussian functions, making it well suited for extended systems such as vacuum slabs and porous materials. GAPW-based all-electron treatments are also available in CP2K for cases where semi-core states or core excitations are relevant. At present, the CP2K linear-response module used in this context does not include *k*-point sampling, so simulations rely on supercell descriptions. First benchmarks on crystalline pyrazine showed that Newton-X/CP2K surface hopping with orbital-overlap couplings can treat dense excited-state manifolds involving 80 excited states, requiring more than 3000 couplings, for an sTDA/PBE setup.^87^

Complementary localized-excitation treatments in molecular crystals are available through the Newton-X interface with fromage.^88^ This implementation is primarily intended for molecular crystals, especially weakly bound ones. In this approach, excited states are computed with a two-layer ONIOM(QM:QM′) setup, and several point-charge embedding schemes are supported, ranging from Ewald embedding to self-consistent alternatives.^88^ Support for covalently bonded molecular crystals through link atoms has also been introduced, although its application to nonadiabatic dynamics remains less explored. In contrast, this interface is not designed for metallic or inorganic semiconducting materials. All electronic-structure methods interfaced in fromage can be used for the QM region with TD-BA couplings, while xTB and DFTB can be used for the QM′ layer.

In these hybrid and extended-system setups, the additional Newton-X overhead is usually small compared with the cost of the electronic-structure or embedding calculation itself. The practical cost is therefore mainly controlled by the size and level of theory of the active electronic region, the number of states and gradients requested, and whether nonadiabatic information is obtained from explicit NACVs, overlaps, or approximate curvature-based couplings. This makes the environmental treatment primarily a modeling and electronic-structure choice, while Newton-X provides the propagation and data-management layer.

Beyond atomistic embedding and extended condensed-phase models, Newton-X also includes open-system extensions. In particular, CS-FSSH implemented in NX-NS enables nonadiabatic dynamics of metastable states through state-specific decay widths, making it suitable for resonance-driven processes such as transient-anion dynamics.^22^ In a related spirit, Skitten implements SLED (Section 3.2), a stochastic Schrödinger extension of Ehrenfest dynamics designed to describe decoherence and dissipation within an open-quantum-system framework.^17^

### 3.6 Nonadiabatic couplings

Electronic-state transitions in MQCD are driven by nonadiabatic couplings, which can be obtained in three main ways. Some electronic-structure methods (*e.g.*, in MRCI and CASPT2) provide nonadiabatic coupling vectors (NACVs), **d**_*MN*_(**R**) ≡ 〈*φ*_*M*_|∇_**R**_*φ*_*N*_〉, where |*φ*_*N*_〉 is the adiabatic electronic state *N* obtained at the nuclear geometry **R**.^89^

Nonadiabatic couplings can also be expressed as time-derivative couplings (TDCs), *σ*_*MN*_(*t*) ≡ 〈*φ*_*M*_|∂_*t*_*φ*_*N*_〉 = **Ṙ**·**d**_*MN*_, which are commonly evaluated from wavefunction overlaps *S*_*MN*_(*t*_1_,*t*_2_) = 〈*M*(*t*_1_)|*N*(*t*_2_)〉 along the trajectory **R**(*t*):^90^24
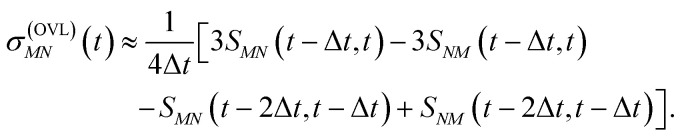


In addition to this finite-difference estimate, Newton-X 26 implements norm-preserving interpolation (NPI),^91^ which constructs a continuous, norm-preserving interpolation of the adiabatic electronic wavefunctions within each nuclear time step and evaluates the TDC from the interpolated evolution. NPI is useful when TDCs are sharply peaked, because the finite time-step sampling may otherwise miss or overestimate the coupling maximum. The overlap matrix **S** also supports local diabatization schemes.^29^

In Newton-X, overlap-based TDCs can be evaluated with either the determinant-derivative (DD) or orbital-derivative (OD) algorithm, depending on the electronic-structure information provided by the interface.^92,93^ In the DD route, electronic-state overlaps are assembled from overlaps between many-electron Slater determinants. In the OD route, the antisymmetric determinant structure is accounted for analytically, reducing the calculation to finite differences of one-electron orbital overlaps. This usually makes OD cheaper than DD.

For methods without explicit many-electron wavefunctions—typically the case of linear-response methods such as TDDFT and ADC(2)—TDCs can be obtained through auxiliary constructions.^92^ For internal conversion to the electronic ground state, however, such approaches must be used with particular caution. In single-reference linear-response descriptions, the topology and dimensionality of the crossing seam with the ground state may be incorrect,^94,95^ making the dynamics unreliable near very small gaps. A common practical strategy is therefore to terminate trajectories when the energy gap falls below a predefined threshold, and to interpret the event as reaching the ground-state decay region rather than attempting to propagate through the crossing. This limitation applies only to crossings involving the reference ground state; crossings between excited states are not affected by this seam-dimensionality problem, although their reliability still depends on the quality of the chosen electronic-structure method.

The CP2K interface implements a distinct overlap-based route for periodic GPW/GAPW calculations.^87^ The overlaps in this case include the periodic repetition of the Gaussian basis and the corresponding lattice-vector sums, so the resulting overlap information is consistent with the periodic electronic-structure calculation. The same CP2K-generated overlap information can then be used to construct overlap-based TDCs for FSSH or for AIMS coefficient propagation, or to define the transformation matrix used in LDSH. Because CP2K obtains TDA excitation amplitudes from a Sternheimer formulation in an atomic-orbital/occupied-space representation, the interface also reconstructs the virtual-orbital components and transforms the amplitudes to the molecular-orbital representation before passing them to Newton-X. The explicit periodic orbital-overlap and amplitude-transformation expressions are given in the dedicated CP2K/Newton-X interface publication.^87^

Finally, TDCs can also be approximated from PES curvature. In the time-dependent Baeck–An (TD-BA) approximation,^18^ which is closely related to the curvature-driven time-derivative coupling (κTDC),^96^ the TDC is estimated at each time step from energy gaps Δ*E*_*MN*_ = *E*_*M*_ − *E*_*N*_ and their time derivatives:25
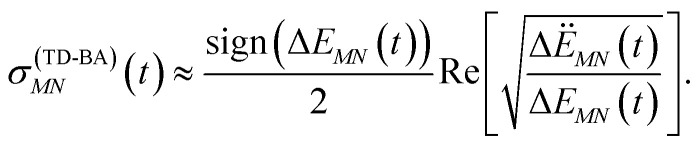
where sign(*x*) = 1 for *x* ≥ 0 and −1 for *x* < 0 ensures the antisymmetry of *σ*_*MN*_ upon state switch, and the second derivative in time is numerically computed during the propagation. Related curvature-based ideas also appear in LZSH (see Section 3.1),^32^ where the local gap profile is used instead to evaluate a Landau–Zener-type hopping probability at local minima of the adiabatic energy gap.

Either with NACVs or TDCs, the couplings inherit an arbitrary phase (sign) from the adiabatic electronic states. In Newton-X, the resulting discontinuities are corrected directly in the NACVs or overlap matrices along the trajectory to avoid spurious sign flips. This is not necessary if the local diabatization scheme is employed.

Curvature-based approaches have become popular in MQCD applications.^38,97–99^ However, because they are derived under Landau–Zener-type, local two-state crossing assumptions,^35^ their use beyond that regime is heuristic and should be validated case by case.

Spin–orbit couplings (SOC) can be obtained through an interface to PySOC,^11^ enabling SOC evaluations within linear-response TDDFT workflows for use in Newton-X spectral simulations. Intersystem crossing MQCD, however, is not currently supported in Newton-X.

Newton-X supports all these coupling options, depending on the electronic-structure method and interface (Table 2). For most molecular interfaces, overlap-based TDCs are computed using the CIOVERLAP program;^100^ in the CP2K interface, the same DD or OD overlap logic is retained, but the underlying atomic-orbital overlap matrix is generalized to the periodic GPW/GAPW representation, still using CIOVERLAP.^87^ This flexibility in the treatment of nonadiabatic couplings is one of the practical strengths of Newton-X, allowing the same dynamics framework to be used across interfaces that provide either native couplings, wavefunction overlaps, or only energy-based approximate couplings.

### 3.7 Machine learning dynamics

Machine-learning (ML) potentials can replace on-the-fly electronic-structure calculations by predicting potential energies, gradients, and, when included in the training targets, nonadiabatic coupling information, thereby accelerating the main bottleneck of MQCD simulations.^101,102^ In a typical ML dynamics workflow, nuclear geometries are labeled with reference electronic-structure data, usually including state energies and their nuclear gradients. The ML model is then trained to reproduce these quantities as functions of the molecular geometry. During dynamics, the trained model provides the energies needed for electronic-state propagation and the gradients required to propagate the classical nuclei. In many neural-network potentials, the gradients are obtained by differentiating the ML-predicted energies with respect to the nuclear coordinates, ensuring a consistent relation between energies and forces.

In practice, ML models are system-specific and require careful sampling of the relevant regions of the high-dimensional configurational space while respecting translational, rotational, and permutational invariances. These requirements are commonly addressed with modern neural-network architectures^103,104^ combined with active learning, in which the training set is iteratively enriched when the model's uncertainty is high.^105–107^ In this strategy, preliminary models are trained from an initial labeled dataset and used to propagate trial dynamics. Geometries for which the model uncertainty is high are then recomputed at the reference electronic-structure level, added to the training set, and used to update the model. Within this framework, ML is not treated merely as a surrogate PES replacement, but as a practical route to extend MQCD simulations toward system sizes, trajectory counts, and better-converged observables that would be difficult to achieve with conventional on-the-fly electronic-structure calculations.

In Newton-X, ML dynamics is orchestrated by the MELTS program,^106^ which loads the Python ML stack once,^108^ coordinates calls to NX-NS and ML models *via* MLatom,^75^ and enables efficient inter-process communication *via* socket-based interfaces.^109^ In the current workflow, MELTS uses the ML-predicted energies and gradients to propagate FSSH trajectories, while time-derivative couplings can be obtained from the predicted energy gaps using the TD-BA approximation. A practical limitation of ML-driven MQCD is that standard ML potentials provide energies and forces but not electronic wavefunctions or densities; therefore, electronic properties are typically computed *a posteriori* for selected geometries using quantum-chemical calculations. Yet, recent applications indicate that the end-to-end cost of active-learning ML MQCD (data generation, training, ML dynamics, and *post hoc* property calculations) can be substantially lower than that of conventional on-the-fly MQCD using quantum-chemical methods.^105–107^

## 4 Analysis, postprocessing, and archiving

Completing the trajectory propagation is only one step toward extracting physically meaningful MQCD results. In practice, the scientific value of MQCD simulations depends just as much on how trajectory ensembles are consolidated, interpreted, and preserved as on how they are propagated. Raw outputs must be curated (*e.g.*, removing duplicates arising from restarts), transformed into observables through statistical analysis, augmented with derived properties from postprocessing, and archived in a form that enables verification and reuse (Fig. 2-right). The following subsections describe how Newton-X supports these steps, from dataset consolidation to long-term preservation.

### 4.1 MQCD data consolidation

The output of an MQCD simulation is an ensemble of trajectories intended to approximate the evolving nuclear wavepacket. Ensemble averages provide time-dependent variables such as state populations and other state-resolved properties as functions of time and nuclear coordinates. These results can be summarized through basic statistics (means, variances) and uncertainty estimates, for example, *via* bootstrap resampling. This analysis step is central because it is where MQCD yields quantitative predictions and enables comparison with experimental observables.

In addition to basic statistics, MQCD datasets often contain higher-level structure: trajectories may separate into distinct pathways or dynamical regimes that differ in reaction coordinate, timescale, or branching ratios.^110,111^ Revealing and quantifying such patterns typically requires transforming the Cartesian geometries into chemically meaningful, symmetry-aware representations.^111^ Examples include internal coordinate sets,^112^ Cremer–Pople–Boeyens ring puckering classification,^113,114^ or pairwise distance matrices, which provide descriptors better suited for statistical exploration of the underlying potential-energy landscape. These representations can then be analyzed using unsupervised learning workflows that combine preprocessing of the trajectory data with dimensionality reduction and clustering techniques to identify groups of trajectories associated with distinct dynamical behaviors.^115,116^

To make this analysis practical and reproducible for large ensembles, Newton-X provides the Ulamdyn program,^111^ which implements a dedicated postprocessing pipeline for MQCD datasets. Through this modular integration, trajectory outputs generated by Newton-X can be automatically curated, transformed into invariant molecular descriptors, and analyzed using unsupervised learning tools, thereby extending the platform beyond conventional geometrical analysis toward data-driven identification of dynamical pathways.

### 4.2 MQCD postprocessing

Beyond data consolidation, MQCD trajectory ensembles can be further postprocessed for several purposes. These include extracting observables that are impractical to compute on the fly, extending the spectroscopic information obtainable from the trajectories, and recovering physical effects not explicitly included in the original dynamics. A common example is the calculation of quantities that are too expensive to evaluate and store during the dynamics. This applies, for instance, to one-electron transition density matrices (1TDMs), which are central for characterizing excited states.^117^ Because 1TDMs are expensive to track during dynamics, they are usually computed *a posteriori* for a selected subset of geometries, retaining the time labels to follow their evolution.^118^

In addition to the analyses available in Newton-X, trajectory outputs can be postprocessed with external tools. One recent example is WaveMixings.jl,^119^ which implements the quasi-classical doorway-window approximation for time-resolved nonlinear electronic spectra and can use NX-NS outputs as input. Such interoperability expands the range of spectroscopic observables that can be extracted from Newton-X simulations.

Postprocessing can also incorporate physical effects absent from the original simulations. One example is excitation under continuous, incoherent illumination (*e.g.*, sunlight), which can drive the system to a nonequilibrium steady state with a constant population flux.^120^ While MQCD typically assumes an instantaneous excitation (often within a narrow energy window, mimicking coherent laser preparation), ensembles generated from broad blackbody initial conditions can be reweighted and combined to model incoherent excitation within the mixed quantum–classical pulse-ensemble (MCQ-PE) approach.^9^

Finally, postprocessing can be used to recover quantum features beyond the original MQCD approximation, such as coherence effects. The QDCT approach,^45^ for example (accessed *via* the Legion program), uses surface-hopping data supplemented by interpolation to integrate a TDSE for Gaussian-dressed trajectories. This strategy aims to achieve the accuracy of more strongly coupled methods at a computational cost closer to that of FSSH and is still in development.

### 4.3 Archiving

MQCD simulations are computationally demanding and can carry a non-negligible carbon footprint,^121^ so archiving trajectories and inputs helps avoid unnecessary recomputation. Publicly sharing MQCD datasets also supports verification and reproducibility in the computational chemistry community.

Newton-X users are strongly encouraged to deposit their data in public repositories with persistent identifiers (*e.g.*, Zenodo) at the time of the first publication reporting the results. Newton-X provides scripts to clean and organize datasets for deposition, following FAIR principles (findable, accessible, interoperable, and reusable).

## 5 Conclusion

Over two decades of development, Newton-X has evolved from a surface-hopping code with flexible links to third-party quantum-chemistry packages into a broader platform for mixed quantum–classical dynamics. Newton-X 26 consolidates this evolution into a modular ecosystem that spans the full MQCD workflow, from spectrum and initial-condition generation to dynamics propagation, data consolidation, postprocessing, and archiving. More broadly, Newton-X 26 illustrates a software design strategy in which trajectory propagation, electronic-structure interoperability, machine-learning acceleration, and data stewardship are treated as separable yet interconnected layers of a reproducible simulation ecosystem.

While surface hopping remains its most mature and broadly developed framework, the platform also supports decoherence-corrected Ehrenfest dynamics and *ab initio* multiple spawning, alongside broad electronic-structure interoperability and compatibility with established workflows. By combining methodological flexibility with workflow-level support for analysis, reproducibility, and data reuse, Newton-X 26 provides a practical platform for both routine MQCD applications and continued method development.

## Author contributions

MB: design and coordination of the Newton-X platform; core development of NX-CS; Skitten development; draft of this manuscript. RSM: core development and maintenance of the Newton-X platform; Legion design and coding; NX-interfaces design and coding; Newton-X documentation. BD: core development of NX-NS. MOB: design and coding of MELTS. MBo: Newton-X/Gaussian QM/MMpol interface. MBr: Newton-X/fromage interface. RCO: Newton-X/fromage interface and previous developments for NX-CS. EGFM: Newton-X/OpenMolcas interface. POD: coordination of Newton-X/MLatom interface. GG: development of methods and core codes in NX-CS. AH: Newton-X/CP2K interface. FH: Newton-X/fromage interface. GI: Newton-X/Gaussian/ONIOM interface: dynamics. RK: multiple-timestep routines. FK: CS-FSSH and importance sampling. BM: Newton-X/Gaussian QM/MMpol interface. HL: initial coordination of NX-CS development. SM: ZPE corrections; PySOC 2 coding; scripting; testing. AM: LVC implementation. FPe: Newton-X/Gaussian/ONIOM interface: initial conditions. MP: development of methods and core codes in NX-CS. MPJ: design and coding of Ulamdyn. JP: CIOVERLAP development. FP: local diabatization implementation; contributions to Columbus and TURBOMOLE interfaces. NR: Newton-X/Gaussian ONIOM interface. ESG: EXASH interface, MOPAC-PI interface. TT: Newton-X/CP2K interface. JMT: Newton-X/OpenMolcas interface; Newton-X/OpenQP interface; scripting; testing. AAT: Skitten development. MTNV: Newton-X/OpenMolcas interface supervision. LV: Newton-X/CP2K and WaveMixings.jl interfaces. All authors contributed to the final manuscript version.

## Conflicts of interest

There are no conflicts to declare.

## Data Availability

No new datasets were generated for this work. The Newton-X platform described in this article is distributed as open-source software and is available free of charge at https://www.newtonx.org.
